# Gut bacterial peptides with autoimmunity potential as environmental trigger for late onset complex diseases: *In–silico* study

**DOI:** 10.1371/journal.pone.0180518

**Published:** 2017-07-05

**Authors:** Sapna Negi, Harpreet Singh, Anirban Mukhopadhyay

**Affiliations:** 1 National Institute of Pathology (ICMR), Safdarjung Hospital Campus, New Delhi, India; 2 Biomedical Informatics Centre, Indian Council of Medical Research (ICMR) Headquarter, New Delhi, India; 3 Department of Genetics, University of Delhi, New Delhi, India; Istituto di Ricovero e Cura a Carattere Scientifico Centro di Riferimento Oncologico della Basilicata, ITALY

## Abstract

Recent evidences suggest that human gut microbiota with major component as bacteria can induce immunity. It is also known that gut lining depletes with ageing and that there is increased risk of autoimmune and inflammatory disorders with ageing. It is therefore likely that both may be correlated as depletion of gut lining exposes the gut bacterial antigens to host immune mechanisms, which may induce immunity to certain bacterial proteins, but at the same time such immunity may also be auto-immunogenic to host. This autoimmunity may make a protein molecule nonfunctional and thereby may be involved in late onset metabolic, autoimmune and inflammatory disorders such as, Diabetes, Rheumatoid Arthritis, Hyperlipidemias and Cancer. In this *in-silico* study we found a large number of peptides identical between human and gut bacteria which were binding to HLA-II alleles, and hence, likely to be auto-immunogenic. Further we observed that such autoimmune candidates were enriched in bacterial species belonging to *Firmicutes* and *Proteobacteria* phyla, which lead us to conclude that these phyla may have higher disease impact in genetically predisposed individuals. Functional annotation of human proteins homologous to candidate gut-bacterial peptides showed significant enrichment in metabolic processes and pathways. Cognitive trait, Ageing, Alzheimer, Type 2 diabetes, Chronic Kidney Failure (CKF), Chronic Obstructive Pulmonary Disease (COPD) and various Cancers were the major diseases represented in the dataset. This dataset provides us with gut bacterial autoimmune candidates which can be studied for their clinical significance in late onset diseases.

## Introduction

Inflammation and autoimmunity is a major factor in almost all late onset diseases, like, Type II Diabetes, Rheumatoid Arthritis, Multiple Sclerosis, Inflammatory Bowel Disorder, Systemic Lupus Erythematosus and Cancer. Of the several mechanisms leading to autoimmunity, molecular mimicry, due to its sequence similarities between foreign and self-peptide, is one such mechanism known to result in cross-activation of pathogen derived autoreactive T or B-cells. These T and B-cell can cross react with host epitopes, thus leading to autoimmunity [1]. Viruses and their peptides are the main non-self-molecules studied for autoimmunity.

Human gut is the connection between environment and human system. It therefore, is the major human reservoir of environmental microbes which are kept isolated from rest of the human system by intestinal mucosal barrier. Loss in this barrier may result in epithelial permeability to gut microbiota which may lead to a phenomenon of molecular mimicry leading to autoimmunity. Many studies have shown expression of MHC class II molecules on intestinal epithelial cells (IEC) and that IECs are capable of processing and presenting luminal peptides and proteins to immuno-competent T-cells [2–4]. Integrity of this mucosal lining depends on factors like, diet, ageing, microbial interactions and disease conditions. In addition, mucosal secretions decreases with age [5–8] and it was also shown that ageing increases colonic permeability [9]. This may lead to exposure of microbial peptides to IEC. Antibodies generated against gut microbial peptides cross reactive to human proteins may lead to depletion in such protein functions, leading to late onset diseases. Metabolic, inflammatory and autoimmune diseases are common in ageing population, and the underlining factor could be antibodies to gut microbiome which is enriched in metabolic orthologous genes. Lately, role of gut microbiota in host immunity has opened up a new frontier of biomedical research with perspective of host-environment interactions.

Detailed mechanistic studies revealed that introduction of a single gut microbiota species, in an autoimmune arthritis mouse model was able to trigger disease development. Although, many studies have postulated that effect of microbiota on the systemic immune response is mediated by circulation of microbiota-derived soluble factors from the gut to periphery [10], the autoimmune arthritis study provided an alternative mechanism where microbiota (composition or microbiota-derived products) can affect the immune system by induction of Th17 cells present on lamina propria of small intestine [11, 12]. Th17 cells then migrate into the peripheral lymphoid tissue and secrete IL-17, which in turn, acts directly on B cells and induces systemic B cell differentiation and antibody production. This ultimately can lead to development of autoimmune disease via molecular pattern recognition from gut microbiota [13]. Gut microbiota has been shown to play role in inflammatory, and autoimmune disorder like Rheumatoid Arthritis in many other studies as well [14, 15] although the mechanism of this association remains obscure. Understanding these mechanisms is crucial for a better treatment efficacy and personalized patient management.

In ageing population and also in inflammatory disease patients, increased levels of serum antibodies to gut bacteria and oral bacteria has been shown [16–19] which strengthens our belief in gut bacteria having antigenic potential. These studies encouraged us to consider gut bacterial peptides homologous to human peptide to be involved in autoimmunity which might be involved in disease etiology by bringing down effective protein quantity. This molecular mimicry to gut bacteria might be the cause of some late onset (if not all) metabolic, autoimmune/inflammatory diseases in genetically predisposed individuals with intestinal permeability. In ageing population this process might be pronounced as the goblet cell’s mucin replenishing ability may decrease and there might be a break-down in cell-cell adhesion junctions in gut [9, 20]. Although, the mechanism of processing of bacterial antigens to raise antibodies is not very clear, autoimmunity to otherwise non-pathogenic commensal bacteria might be possible in an ageing population. Exploring the role of gut microbes in intestinal as well as extra-intestinal diseases will significantly advance our understanding of disease pathogenesis and may further help develop strategies for a therapy based on controlling autoimmunity to gut microbiota.

In our understanding, this is the first instance where an *in-silico* study was carried out to know if gut bacterial peptides has potential to raise antibodies against human proteins. Such peptides could be auto-immunogenic to humans. The auto-antigenicity towards a specific tissue protein may be involved in particular disease pathology depending on the tissue involved. Herein, human proteins cross-reacting to antibodies against candidate peptides were looked for their tissue specific expression, biological processes and to the diseases they are associated with. This approach has given a comprehensive view of plausible effects of autoimmunity raised to the gut candidate peptides on human system.

## Methods

**Fig 1:**
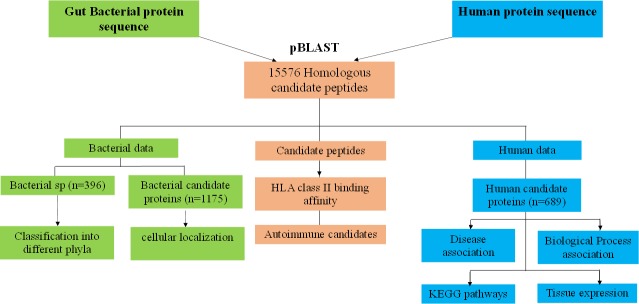
Methodology adopted for identifying gut bacterial peptides with auto-immunity potential (candidate peptides) and their functional annotation.

Methodology adopted for identifying gut bacterial peptides with auto-immunity potential is depicted in Fig 1 and has also been uploaded at PLOS ONE’s site (https://www.protocols.io/view/gut-bacterial-peptides-with-autoimmunity-potential-ibccaiw).

### Candidate peptides identification and characterization

#### Identification of candidate peptides

Sequence identity search was conducted between all gut bacterial species/genus and complete human proteome. Here we took gut bacterial species/genus information from Human Microbiome Project database (www.http://hmpdacc.org/) and used all available annotated protein sequences of these bacterial species/genus from Uniprot database (https://www.ebi.ac.uk/uniprot). Wherever, gut bacterial species information was mentioned in HMP database, the related species were looked for sequence homology with human proteins. 319 bacterial strain (from a total of 823 in HMP) found in gut, did not have species information but had genus information. Protein sequences of all the species of these genera were searched against human protein database (Uniprot database). Sequence similarity search using pBLAST (Basic Local Alignment Search Tool for protein sequences) was carried out, to get peptide similarity between gut bacteria and human expressed proteins. Data on length and sequence of homologous regions, and protein/gene IDs and names were recorded. Peptides having homologous regions of ≥9 aa were included in the study.

All the gut bacterial proteins as well as human proteins corresponding to homologous peptides were subjected to various characterization in order to further understand their biological relevance to disease etiology.

### Bacterial characterization

Bacterial proteins were studied for their antigenic potential and for location of peptides in bacterial cell. This may help us in predicting effect of antigen on our immune system. Predominant gut bacterial phyla possessing these auto-immune peptides were then estimated in the dataset.

#### Candidate peptide antigenic potential

**Fig 2:**
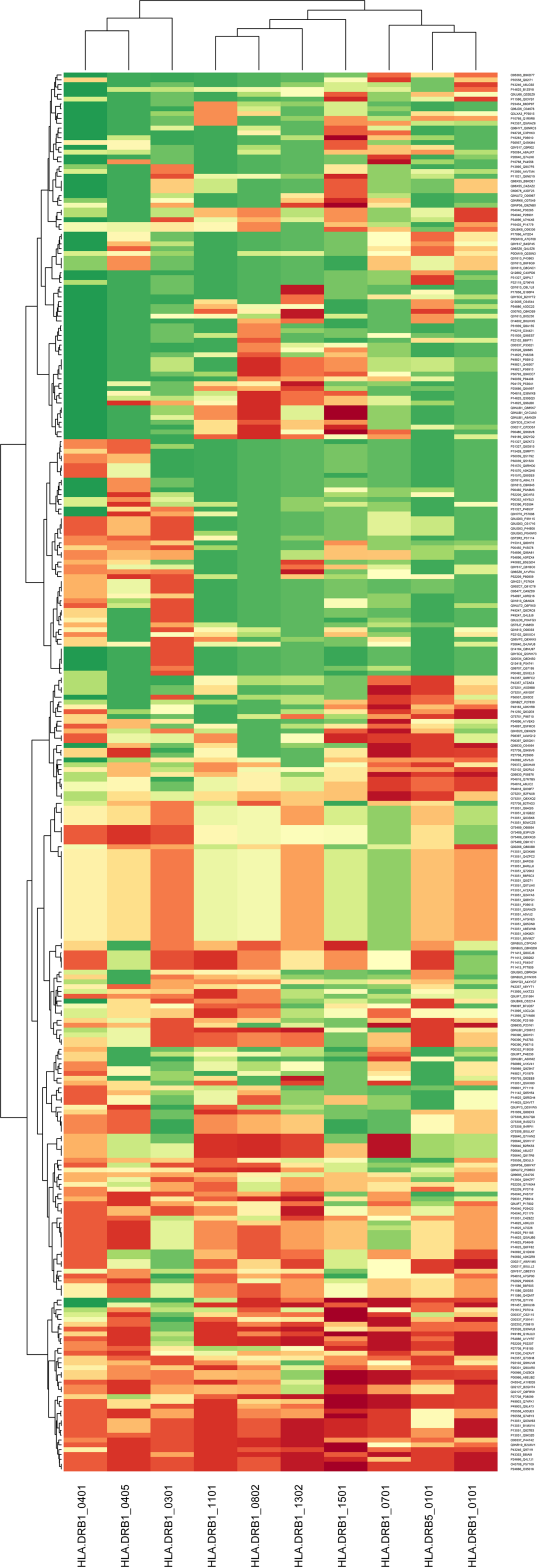
Clustered heatmap of human candidate proteins associated with late onset complex diseases and their binding affinity threshold with common HLA class II alleles. Binding affinity threshold [range: 1(red)– 11(green)]. Lower the threshold higher is the binding affinity with particular HLA class II allele. Human candidate proteins and the homologous gut bacterial proteins (Hu. protein_Bac. Protein, as provided in S1 Table) have been indicated on the y-axis. The common HLA class II alleles tested have been indicated on x-axis. Only, human candidate proteins depicted in Fig 2 and having common HLA class II binding affinity are represented here.

The homologous peptides were subjected to HLA class II binding profile using *in-silico* method. The peptide candidates (as seen in S1 Table**)** were analyzed for HLA class II using ProPred software [21, 22]. Propred is a matrix-based method that allows prediction of MHC binders for various alleles based on experimental binding profiles. Binding scores were generated for 50 HLA class II (HLA-II) alleles. Affinity values were generated for candidate as well as random peptides from the same protein. The threshold binding values for all peptides were subjected for significant association using non-parametric Mann-Whiteny test. A significant difference in binding threshold values defines a difference in binding to candidate peptide than to others from same protein with respect to presence of specific HLA-II allele in the host. However, all the peptides (irrespective of their significant binding to HLA) were considered for further analysis for their role in human diseases. The candidate peptides having association of corresponding human protein with common diseases are displayed in heatmap (Fig 2) to show their binding affinity with various HLA-II alleles.

#### Bacterial protein location

The set of unique candidate peptides were subjected to PSLpred [23] server for identification of bacterial candidate protein’s cellular location, that is, extracellular, periplasmic, outer membrane, inner membrane or cytoplasmic.

#### Predominant gut bacterial phyla in dataset

The bacterial species encoding candidate peptides were subjected for phylum level classification (www.microwiki.com) to know predominance of each bacterial phylum in our dataset of autoimmune candidate peptides. Difference in number of species in each phylum was compared with number of autoimmune candidates encoded by them. P-value and 95% CI for a significant difference was calculated using pearson’s correlation using SISA statistical tool (www.quantitativeskills.com/sisa/).

### Human protein characterization

The human proteins corresponding to candidate peptides (candidate human proteins) were characterized using systems biology approach to know which biological process, KEGG pathway, and tissue may be affected and the type of diseases associated, with raising of antibody to the candidate peptide.

#### Disease association

**Fig 3:**
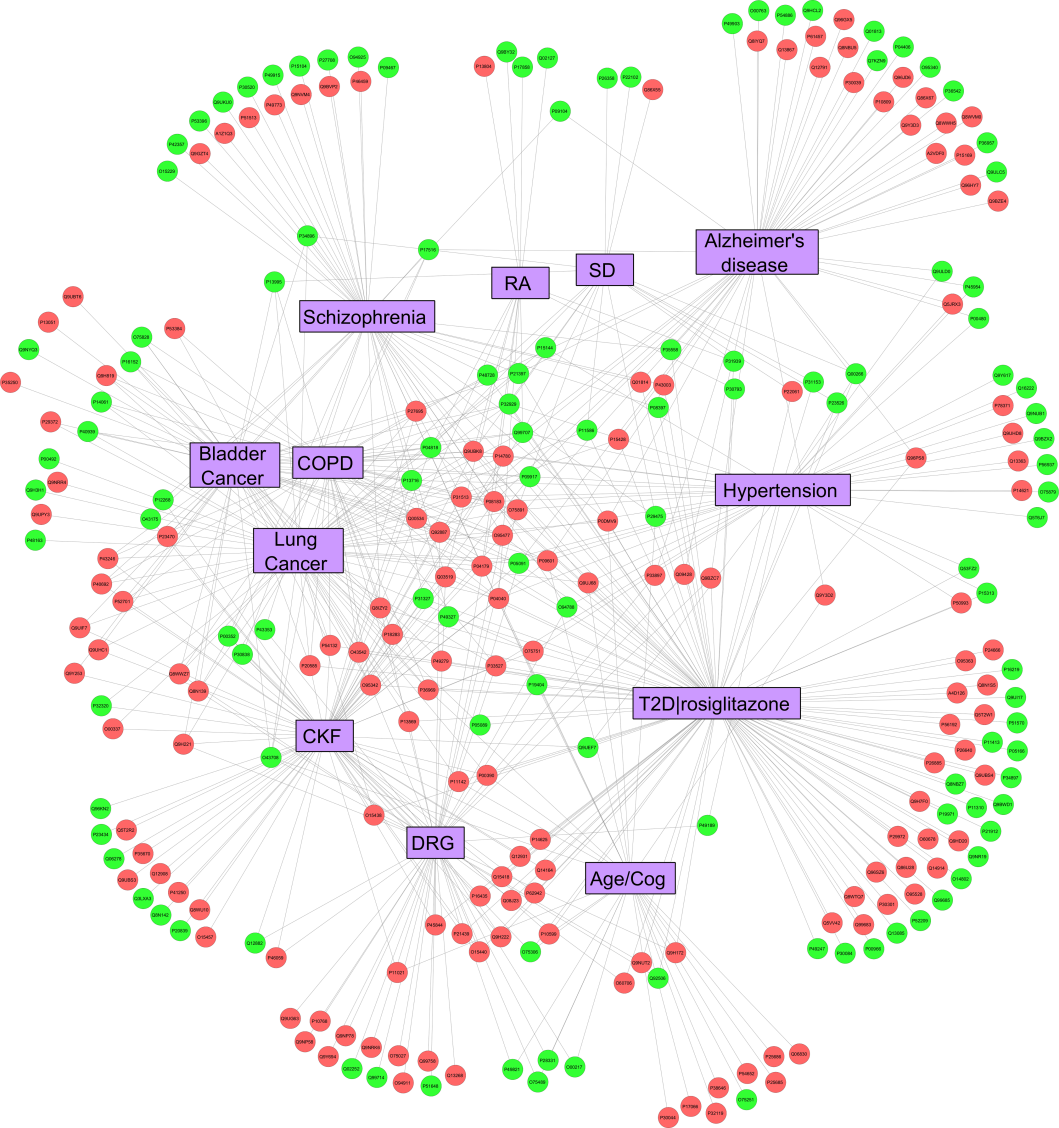
Cytoscape network displaying distance relationship of late onset complex diseases (purple nodes) associated with human candidate proteins (circular nodes). Shorter the distance between disease node and human candidate protein node higher are the number of gut bacterial species possessing autoimmune candidate peptide targeting corresponding human candidate protein. Green circular nodes are metabolic genes as defined by KEGG metabolic pathway.

575 human proteins were annotated under disease subgroups using the Genetic Association Database (GAD). Genes were classified using GAD module of DAVID functional annotation tool (https://david-d.ncifcrf.gov/summary.jsp). To know diseases with significant number of associated candidate human proteins matching our list, p-value was generated using boneferroni multiple correction method. A distance matrix to disease was created based on number of gut bacterial species having the candidate peptide with autoimmunity potential. Closer the peptide to the disease in the cytoscape (Fig 3) higher is the number of bacterial species harboring the specific candidate peptide.

#### Tissue specificity

The details on tissue specific expression of candidate human proteins was generated using Uniprot tissue database (UP_Tissue database) and p-values were calculated through DAVID functional annotation tool. Boneferroni multiple correction was used to get adjusted p-values. A total of 667 candidate human proteins could be annotated for tissue expression.

#### Gene ontology and functional annotation

Candidate human proteins were studied for their involvement in biological processes using DAVID functional annotation tool. These genes were also studied for their involvement in molecular pathways using KEGG database and significant pathway associations were recorded. All the p-values were checked for multiple corrections using boneferroni. Total 670 and 470 candidate human proteins were annotated by biological processes and KEGG pathways respectively.

## Results

### Candidate peptides identification and HLA class II binding affinity

#### Identification of candidate peptides

Gut bacterial peptides with ≥9 amino acids length and having human homology were selected as candidate peptides for further analysis (henceforth denoted as candidate peptides). 15576 candidate peptides (unique candidate peptides = 2295) from 396 unique bacterial species were found to have peptide homology with 689 unique human proteins (candidate human proteins). Information was recorded on their sequence, protein and gene Ids (both human and bacterial) and bacterial species having the peptide (S1 Table**)**.

#### Binding potential of candidate peptides with HLA-II targets

All 15576 peptides with their binding threshold scores and comparative binding threshold scores for random peptide from the same protein has been listed in S2 Table. Binding threshold of ≤ 3 was considered as peptide binding affinity to HLA-II alleles. The threshold scores to common HLA-II alleles for peptides linked with candidate human proteins associated with important diseases have been displayed in Fig 2.

Of the total 50 HLA-II alleles tested 35 alleles significantly differed in their binding to candidate peptides than to a random non-homologous peptide from the same protein **(**S1 Fig**)**. None of the HLA-II alleles were uniformly binding to all the peptides (S2 Table) and therefore, would raise antibodies specific to peptides, if presented with the antigen (peptide). 24 gut bacterial peptides with homology to four human peptides from proteins like (Low molecular weight phosphotyrosine protein phosphatase, Aldehyde dehydrogenase family 3 member B1, Maleylacetoacetate isomerase and Uracil-DNA glycosylase) were found to have binding affinity with either of the ten common HLA-II alleles [24] namely, HLA-DRB1_0101, HLA-DRB1_0301, HLA-DRB1_0401, HLA-DRB1_0405, HLA-DRB1_0701, HLA-DRB1_0802, HLA-DRB1_1101, HLA-DRB1_1302, HLA-DRB1_1501 and HLA-DRB5_0101 (S2 Table).

### Bacterial candidates characterization

#### Predominant gut bacterial phyla

The predominant bacterial phylum in dataset with autoimmune candidate peptides was *Proteobacteria* and *Firmicutes*. On the other hand *Actinobacteria*, *Bacteroidetes and Fusobacteria*, were under-represented in this dataset. *Verrucomicrobia*, *Synergistetes*, *Enterobacteria* and *Tenericutes* were found to be very low in HMP gut dataset itself and so were also either absent or under-represented in autoimmune candidate dataset. Bacterial species belonging to *Actinobacteria* phylum was found to be significantly (p = 0) low in autoimmune candidate peptides (**Table 1**). Whereas, *Proteobacteria* (not significant) and *Firmicutes* (p = 0.04) were having more of such peptides.

**Table 1:** Number of bacterial species encoding autoimmune peptides in each gut bacterial phyla of the autoimmune candidate peptides dataset.

Phylum	Bacterial species among autoimmune candidates [n, (proportion)]	Autoimmune candidates [n, (proportion)]	p-value	95% CI
*Actinobacteria##*	62 (0.16)	628 (0.04)	0	0.195–0.341
*Bacteroidetes*	9 (0.02)	272 (0.02)	NS	
*Firmicutes#*	146 (0.37)	7012 (0.45)	0.04	1.002–1.326
*Fusobacteria*	3 (0.008)	58 (0.004)	NS	
*Proteobacteria*	175 (0.44)	7567 (0.49)	NS	
*Verrucomicrobia*	1 (0.002)	19 (0.001)	NS	

Significantly larger number of bacterial species is required from *Actinobacteria* phyla for encoding autoimmune peptides. *Proteobacteria* and *Firmicutes* phylum on the other hand require lower number of bacterial species to encode for autoimmune peptides and therefore a rise in species belonging to *Proteobacteria* and/or *Firmicutes*, may have a larger impact on host autoimmunity. Overall representation of other phylum (*Bacteroidetes*, *Fusobacteria*, *Verrucomicrobia*, *Synergistetes*, *Tenericutes and Enterobacteria)* are low or absent in our autoimmune dataset.

“##” Significant p-value on multiple correction.

“#” Significant p-value <0.05.

“NS” non-significant

#### Bacterial peptide cellular location

Out of a total unique gut bacterial candidate peptides, 3 were observed to be present on outer membrane, 60 on inner membrane, 15465 are cytoplasmic proteins, 34 were periplasmic and 14 were extracellular (secretory) proteins **(**S3 Table**)**. Selected peptides (based on their importance in common diseases and binding with common HLA-II alleles) were all found to be cytoplasmic (data not shown). Extracellular/secretory proteins listed here may have increased chances of crossing intestinal barrier under conditions of intestinal permeability, and therefore can cause disease pathogenesis. Of the 14 extracellular peptides 6 peptides have binding affinity with HLA class II alleles (S3 and S2 Tables).

### Human candidate protein characterization

Candidate human proteins are human proteins having peptide homology to respective bacterial peptides.

#### Human disease targets

The candidate human proteins were found to be significantly associated with common complex human diseases. 575 candidate proteins were found to have genetic association with diseases. The diseases significantly associated are, Drug Response (DRG), Cognitive trait, Ageing, Spinal Dysraphism (SD), Chronic Kidney Failure (CKF), Chronic Obstructive Pulmonary Disease (COPD) and certain Cancers apart from some other common diseases namely, Type 2 Diabetes (T2D), Hypertension, Alzheimer’s Disease, Rheumatoid Arthritis (RA), Schizophrenia, etc. AIDS, despite of a very significant p-value, was not considered as it contains several other disease associations within it. Many of these proteins were found to be metabolic in function (n = 118, without considering genes related to AIDS) and these metabolic proteins were associated with almost all the diseases (Fig 3 and S4 Table).

#### Human tissue targets

Out of 689 unique candidate human proteins, a total of 667 proteins were classified for specific expression in human tissues. Significant number (boneferroni p-value <0.05) of candidate proteins were associated with tissues like liver, Cajal-Retzius cell, fetal brain cortex, kidney, muscle, adipocyte and lung. Maximum number of candidate proteins were expressed in liver (Table 2 and S5 Table).

**Table 2:** Human candidate proteins and their expression in different tissues.

Tissue	No. of candidate proteins expressed	Percentage of proteins from total annotated to the category	Pvalue	Bonferroni
Liver	169	25	5.27E-23	1.23E-20
Cajal-Retzius cell	40	6	4.09E-16	1.03E-13
Fetal brain cortex	33	5	2.21E-09	5.14E-07
Muscle	62	9	1.28E-06	2.98E-04
Kidney	86	12	4.36E-05	0.0101
Adipocyte	8	1	6.53E-05	0.0151
Lung	136	20	9.90E-05	0.0228
Skin	99	14	2.35E-04	0.0533
Erythrocyte	6	1	0.0016	0.3078
Colon	66	10	0.002	0.3792
Placenta	154	22	0.0036	0.5717
Bones	4	1	0.0075	0.8285
Platelet	32	5	0.014	1
Macrophage	5	1	0.014	1
Small intestine	21	3	0.0249	1
Urinary bladder	15	2	0.03361	1
Eye	53	8	0.0351	1
Skeletal muscle	30	4	0.0415	1

#### Human biological processes and KEGG pathways

Metabolic processes (such as, carbohydrate, lipid, folic acid metabolism, nucleotide and vitamin biosynthesis) and biological processes involved in response to drug, protein folding, translation and oxidation reduction were the significant biological processes associated with candidate human proteins. Of the entire proteins in the carbohydrate metabolic process and mismatch repair category 4.64% and 1.45% respectively were represented in our dataset (Table 3 and S6 Table). KEGG pathway annotated 470 proteins of the dataset and showed metabolic pathway as the most significant pathway which included Glycolysis / Gluconeogenesis, Citrate cycle (TCA cycle), Fatty acid degradation, ABC transporters etc. (Table 4 and S7 Table).

**Table 3:** Human candidate proteins and their significant association with different biological processes.

Biological Process	No. of Candidate Proteins	% of proteins annotated to the category	Pvalue	Bonferroni
oxidation-reduction process	105	15.24	4.16E-39	9.35E-36
tRNA aminoacylation for protein translation	25	3.63	5.74E-24	1.29E-20
protein homotetramerization	24	3.48	2.43E-17	5.46E-14
tricarboxylic acid cycle	18	2.61	4.25E-17	9.56E-14
gluconeogenesis	19	2.76	1.99E-14	4.5E-11
glycolytic process	17	2.47	3.82E-14	8.59E-11
glutamine metabolic process	13	1.89	1.36E-12	3.07E-09
carbohydrate metabolic process	32	4.64	1.93E-12	4.35E-09
glyoxylate metabolic process	14	2.03	3.66E-12	8.24E-09
response to drug	42	6.10	7.24E-12	1.63E-08
transmembrane transport	37	5.37	1.03E-11	2.33E-08
nucleobase-containing compound metabolic process	17	2.47	3.31E-11	7.46E-08
canonical glycolysis	13	1.89	8.39E-11	1.89E-07
ATP metabolic process	14	2.03	9.80E-11	2.21E-07
metabolic process	29	4.21	1.18E-10	2.65E-07
pyruvate metabolic process	12	1.74	1.75E-10	3.94E-07
pentose-phosphate shunt	9	1.31	9.04E-10	0.00000203
nucleobase-containing small molecule interconversion	12	1.74	9.91E-10	0.00000223
purine ribonucleoside monophosphate biosynthetic process	9	1.31	6.57E-09	0.0000148
mitochondrial translation	13	1.89	7.54E-09	0.000017
fatty acid biosynthetic process	14	2.03	8.58E-08	0.000193
purine nucleotide biosynthetic process	8	1.16	1.03E-07	0.000231
nucleoside diphosphate phosphorylation	9	1.31	1.87E-07	0.00042
protein folding	25	3.63	2.11E-07	0.000475
glucose metabolic process	15	2.18	3.07E-07	0.00069
nucleoside metabolic process	9	1.31	3.12E-07	0.000701
phosphorylation	18	2.61	3.97E-07	0.000893
long-chain fatty-acyl-CoA biosynthetic process	12	1.74	5.12E-07	0.00115174
negative regulation of inclusion body assembly	7	1.02	7.16E-07	0.00161009
protein refolding	8	1.16	7.51E-07	0.0016893
one-carbon metabolic process	10	1.45	1.62E-06	0.00362899
pyrimidine nucleoside salvage	7	1.02	2.94E-06	0.00659774
folic acid metabolic process	8	1.16	3.35E-06	0.00750085
cholesterol efflux	9	1.31	3.61E-06	0.00807881
fructose 6-phosphate metabolic process	6	0.87	5.01E-06	0.01121161
branched-chain amino acid catabolic process	8	1.16	5.12E-06	0.01144613
GTP biosynthetic process	7	1.02	5.28E-06	0.01180881
mismatch repair	10	1.45	6.67E-06	0.0148935
fatty acid beta-oxidation	11	1.60	6.85E-06	0.01528487
nucleoside triphosphate biosynthetic process	7	1.02	8.93E-06	0.01988765
heme biosynthetic process	8	1.16	1.10E-05	0.02447202
ADP biosynthetic process	5	0.73	1.21E-05	0.0268425
regulation of translational fidelity	7	1.02	1.44E-05	0.03183529
anion transmembrane transport	9	1.31	1.63E-05	0.0361178
biosynthetic process	9	1.31	1.63E-05	0.0361178
purine nucleotide metabolic process	6	0.87	2.11E-05	0.04634993

**Table 4:** Human candidate proteins and their significant association with different KEGG pathways.

KEGG pathway	No. of Candidate Proteins	% of Proteins annotated to the category	Pvalue	Bonferroni
Metabolic pathways	278	40	1.68E-97	3.84E-95
Biosynthesis of antibiotics	105	15	2.50E-67	5.72E-65
Carbon metabolism	66	10	1.72E-47	3.94E-45
ABC transporters	35	5	7.79E-32	1.78E-29
Biosynthesis of amino acids	40	6	9.72E-27	2.23E-24
Pyruvate metabolism	30	4	6.44E-26	1.47E-23
Glycolysis / Gluconeogenesis	37	5	3.81E-25	8.72E-23
Aminoacyl-tRNA biosynthesis	32	5	1.50E-19	3.43E-17
Valine, leucine and isoleucine degradation	27	4	6.75E-19	1.55E-16
Propanoate metabolism	20	3	1.69E-16	5.08E-14
Glycine, serine and threonine metabolism	23	3	2.02E-16	5.08E-14
Citrate cycle (TCA cycle)	19	3	2.76E-14	6.31E-12
Pentose phosphate pathway	18	3	2.52E-13	5.78E-11
Fatty acid degradation	20	3	1.28E-11	2.93E-09
Glyoxylate and dicarboxylate metabolism	16	2	1.96E-11	4.49E-09
Cysteine and methionine metabolism	17	2	8.71E-10	2.00E-07
Alanine, aspartate and glutamate metabolism	16	2	2.20E-09	5.04E-07
Fatty acid metabolism	18	3	6.14E-09	1.41E-06
Arginine biosynthesis	12	2	1.20E-08	2.76E-06
One carbon pool by folate	12	2	1.20E-08	2.76E-06
Fatty acid biosynthesis	10	1	1.59E-08	3.64E-06
Purine metabolism	35	5	1.75E-08	4.01E-06
beta-Alanine metabolism	14	2	3.67E-08	8.39E-06
Butanoate metabolism	13	2	5.66E-08	1.30E-05
Histidine metabolism	12	2	8.04E-08	1.84E-05
Arginine and proline metabolism	16	2	5.89E-07	1.35E-04
Pyrimidine metabolism	22	3	4.81E-06	0.0011
Drug metabolism—other enzymes	14	2	7.03E-06	0.0016
Tryptophan metabolism	13	2	8.05E-06	0.0018
Selenocompound metabolism	8	1	6.78E-05	0.0154

Human candidate proteins are human proteins having peptide homology to respective bacterial peptides.

## Discussion

Function of the immune system, and so does autoimmunity, is affected by various factors like, host genetics, age and diet. Age of an individual also defines integrity of mucosal layer which is a barrier between host and his microbiota. Microbiota composition is defined by host immunity and vice-versa [25]. Development of immunity, after anergy is established in host, may lead to autoimmune reactions if developed against own functional proteins. Genetically predisposed individuals to autoimmunity are the ones having HLA-II allele that may induce immunity to self-tissue / proteins. Autoimmunity can be raised to certain pathogenic bacteria and also to microbiota [26], through molecular mimicry. As gut is the major source of host interaction with environmental microbes it may be one of the major factors in autoimmunity due to host microbiota in predisposed individuals.

As it has recently been proven in *in-vitro* as well as *in-vivo* studies that gut microbiota has the potential to induce lymphocytes for antibody production and also to raise IgG antibodies [27, 28], this study is well in time to delineate gut bacterial peptides with auto-immunity potential. Through our *in-silico* study we have tried to delineate gut bacterial peptide candidates having antigenic properties to raise auto-antibodies, in individuals with HLA-II binding affinity to these peptides (genetically predisposed individuals). Here we have also delineated HLA-II alleles that could be a risk factor (i.e. make an individual genetically predisposed) to certain late onset diseases. If we know which gut bacterial composition is low in peptides similar to self and thereby is not a potential autoimmunity trigger, then we can use this information as a preventive therapy in genetically predisposed individuals. This may be achieved by means of antibiotics and/or pre/probiotics. Modulation of gut microbiota is predicted to be possible therapy in some autoimmune disorders [29]. Gut microbial composition has also been associated lately with extra-intestinal inflammatory conditions like Rheumatoid Arthritis (RA) [14].

In the present study we have found 15576 gut bacterial peptides (S1 Table) having potential to induce autoimmune response in genetically predisposed host (host with compatible HLA-II allele). The common HLA-II alleles show a binding affinity with 24 candidate peptides (binding score <3, S2 Table) encoded by four different human proteins. These four human proteins were found to be associated with diseases such as cancers, COPD, chronic renal failure and T2D and Atherosclerosis (S4 Table). The most common gene binding to common HLA-II alleles is “aldehyde dehydrogenase 3 family member B1” produced by many E. coli strains. This gene encodes isozyme of aldehyde dehydrogenases and may detoxify aldehydes generated by alcohol metabolism and lipid peroxidation. It may therefore play a role in protection from oxidative stress. As it is an isozyme, its function is not indispensible and therefore, autoantibody to this protein may not result in a specific disease but may reduce the efficiency of protection from oxidative stress. There were 70% of HLA-II alleles tested, significantly displaying different binding affinity to random peptides taken from same protein (S1 Fig), indicating that these HLA-II alleles may have a role in host-microbe interaction leading to autoimmunity.

On comparing diversity within phyla in our dataset it was found that overall representation of bacterial species in *Firmicutes* and *Proteobacteria* phylum is significantly high in our dataset (with autoimmune candidate peptides) (Table 2). This indicates that a control in diversity and amount of *Firmicutes* and *Proteobacteria* may in general have beneficial effect on autoimmune conditions developed due to cross-reactivity to gut bacteria, if any. *Proteobacteria* is the most diverse bacterial phyla and has been linked to dysbiosis and increased risk of diseases [30]. In our dataset, proteins associated with T2D were observed to be over-represented in *Proteobacteria* phyla and therefore a control in *Proteobacteria* phyla may be beneficial for T2D. *Proteobacteria* was also observed to be over-represented in T2D in other studies [31, 32]. This also suggests a plausibility that autoimmunity to metabolic proteins due to the dysbiosis in *Proteobacteria* and/or gut permeability to *Proteobacteria* may be the underlining cause of T2D etiology in some. On the other hand, an increase in *Actinobacteria* might be harmless/beneficial for autoimmune condition raised due to gut bacteria.

Diseases significantly associated (p<0.05) with candidate peptides are mainly those with late age of onset and are multifactorial (genetic as well as environmental factors) in its etiology, like those involving Cognitive trait/Ageing, Cancer, Chronic Obstructive Pulmonary Diseases (COPD) and Chronic Renal Failure (CRF) (Fig 3) except for Spinal Dysraphism (SD). Prevalence of all these diseases have been observed to increase from 2005 to 2015 by a factor almost 2 times [33], which could be due to increase in ageing population. This may necessitates exploration of additional age related factors into the disease etiology such as, decrease in mucous secretion by epithelial goblet cells [5]. SD has an occurrence in early phase of life (i.e. fetal development), but in here it was found that nine candidate peptides (P11586, P31939, P22102, P13995, P04818, P48728, P34896, O75891, Q99707) associated with SD are from ‘One carbon pool by folate’ KEGG pathway. This pathway is involved in folate uptake and so autoimmunity to these peptides in mothers, may lead to a defect in maternal folate uptake, and may induce SD in the fetus **(**Fig 3, Table 4 and S7 Table**)**. Other major associated diseases displayed in Fig 3 are also multifactorial and late in onset.

T2D and response to rosiglitazone, shows a good trend towards association (unadjusted p = 0.05) with 16.4% of total T2D/edema/rosiglitazone associated genes present in our dataset. T2D is known to be a multifactorial condition with elusive causal factor, therefore, autoimmunity to gut bacteria is worth exploring in this disease. T2D is known to be a metabolic disorder and bacterial genome mainly encodes metabolic proteins. Our dataset of potential autoimmune peptides from gut bacteria is predominated by conserved metabolic proteins, and may therefore have an implication in auto-antibody response specifically to host metabolic proteins.

This indicates that if an autoimmunity developed to gut bacterial peptide is cross reactive to human protein, it might have an influence on health and disease. If proven to be the case, autoimmunity to gut bacterial peptides could be a phenomenon in late onset disease etiologies, which needs to be targeted for an effective therapy.

The tissue with maximum cross-reactivity with gut bacterial peptides, is liver followed by cajal-retzius cell, fetal brain, kidney, adipocyte and lung. Autoimmunity to tissues may cause inflammation leading to diseases involving these tissues. For example: proinflammatory markers has been observed in adipocytes of obese individual with insulin resistance [34]. In our dataset also common candidate human proteins have been observed between adipocyte and T2D (Q13085, P24666, P05091). Likewise, there are some overlapping genes between lung tissue and COPD in our dataset as well as between kidney tissue and Chronic Renal Failure; and between fetal brain tissue and Cognitive trait and Spinal Dysraphism (S3 and S5 Tables). Among molecular pathways Metabolic (such as, Glycolisis/Gluconeogenesis) and ABC transporter pathways are the most significant pathways sharing proteins from the dataset (S6 Table). This indicates importance of proteins involved in metabolic pathways in diseases linked to candidate gut peptides. This study suggests that bacterial peptides rather than bacterial species driven analysis might have more relevance of microbiota to late onset complex diseases.

Hallmarks of autoimmunity have recently been associated with many of the late onset complex diseases, like Diabetes (LADA form of diabetes), Macular Degeneration, Rheumatoid Arthritis, COPD etc. [35–38]. We can take evidence from published literature or clinical observations of autoimmunity linked with above mentioned disease pathogenesis and look whether the underlining cause of autoimmunity lies within gut bacterial peptides. The *in-silico* data provided here could be a lead in such directions specifically in diseases those are late in onset.

## Conclusions

In this *in-silico* study we have adopted an approach to identify gut bacterial peptides with potential to raise autoimmunity to the host. We found a large number of gut bacterial peptides which are homologous to human peptides and also binding to HLA-II alleles. Thus, these peptides can stimulate autoimmunity via antibody production to gut candidate peptides. This particularly may occur in aging individuals with depleting mucosal gut lining. Provided that gut permeability being a general phenomenon in ageing population this process may trigger immune responses towards the commensal peptides and contribute to a sustained autoimmunity against human homologous proteins, if remain untreated. This theory seems plausible and needs to be further strengthened by experimentally confirming autoimmunity to commensal gut bacteria. Some interesting observations on correlation of human proteins corresponding to candidate peptides with human diseases and tissues are observed and presented here. More specifically the authors found an association of candidate human proteins with important metabolic processes. Metabolism, is the major process in humans, which gets affected in a genetically predisposed ageing population. Is the underlining cause being gut bacterial antibodies which are cross-reactive to host metabolic proteins? remains to be answered. If so the therapy to this may be preventive in nature which may involve repair of gut mucosal barrier. For the diseased cases depending on genetic predisposition (HLA-II allele) of the individual, the individual may be screened and treated with personalized therapy based on presence and type of antibody. Given the fact that the two phylum (*Proteobacteria and Firmicutes*) found to be associated with T2D, were also observed to have higher proportion of autoimmune peptides in this study, the present study is promising to be taken up for clinical findings. Finally, we would like to conclude that gut bacterial peptides could be the so called environmental trigger to late onset complex diseases in genetically predisposed individuals. Although, the findings of this study using mega data contributes towards understanding of molecular mechanisms which may underline late onset diseases, the present study is an *in-silico* study and needs further experimental confirmation.

## Supporting information

S1 Fig HLA class II alleles showing significant difference in binding affinity between autoimmune candidate peptides and random peptides. (DOCX)

S1 Table Information on gut bacterial peptides having homology to human peptides (candidate peptide list). (XLSX)

S2 Table Binding affinity threshold score of candidate peptides with HLA class II alleles. (XLSX)

S3 Table Location of bacterial candidate proteins on bacterial cell. (XLS)

S4 Table Diseases associated with human candidate proteins. (XLS)

S5 Table Tissue expression of human candidate proteins. (XLS)

S6 Table Biological processes significantly associated with human candidate proteins. (XLSX)

S7 Table KEGG pathways associated with human candidate proteins. (XLS)

## References

[1] Kohm AP, Fuller K.G and Miller S.D. Mimicking the way to autoimmunity: an evolving theory of sequence and structural homology. Trends in Microbiology. 2003; 11(3): 101–105. 12648936 10.1016/s0966-842x(03)00006-4

[2] Van Niel G, Mallegol J, Bevilacqua C, Candalh C, Brugière S, Tomaskovic-Crook E et al Intestinal epithelial exosomes carry MHC class II/peptides able to inform the immune system in mice. Gut. 2003; 52(12): 1690–1697. 14633944 10.1136/gut.52.12.1690 PMC1773888

[3] Mallegol J, Van Niel G, Lebreton C, Lepelletier Y, Candalh C, Dugave C et al T84-intestinal epithelial exosomes bear MHC class II/peptide complexes potentiating antigen presentation by dendritic cells. Gastroenterology. 2007; 132(5): 1866–1876. doi: 10.1053/j.gastro.2007.02.043 17484880 10.1053/j.gastro.2007.02.043

[4] Bär F, Sina C, Hundorfean G, Pagel R, Lehnert H, Fellermann K et al https://www.ncbi.nlm.nih.gov/pubmed/?term=B%C3%BCning%20J%5BAuthor%5D&cauthor=true&cauthor_uid=23574324. Inflammatory bowel diseases influence major histocompatibility complex class I (MHC I) and II compartments in intestinal epithelial cells. ClinExpImmunol. 2013; 172(2): 280–289. 10.1111/cei.12047 PMC3628330 23574324

[5] Merchant HA, Liu F, Orlu Gul M, Basit AW. Age-mediated changes in the gastrointestinal tract. Int J Pharm. 2016; 512(2): 382–395. doi: 10.1016/j.ijpharm.2016.04.024 27085646 10.1016/j.ijpharm.2016.04.024

[6] Dambroise E, Monnier L, Ruisheng L, Aguilaniu H, Joly JS, Tricoire H et al https://www.ncbi.nlm.nih.gov/pubmed/?term=Rera%20M%5BAuthor%5D&cauthor=true&cauthor_uid=27002861. Two phases of aging separated by the Smurf transition as a public path to death. Sci Rep. 2016; 6: doi: 10.1038/srep23523 10.1038/srep23523 PMC4802314 27002861

[7] Man AL, Gicheva N, Nicoletti C. The impact of ageing on the intestinal epithelial barrier and immune system. Cell Immunol. 2014; 289(1–2): 112–118. doi: 10.1016/j.cellimm.2014.04.001 24759078 10.1016/j.cellimm.2014.04.001

[8] Rera M, Clark RI, Walker DW. Intestinal barrier dysfunction links metabolic and inflammatory markers of aging to death in Drosophila. Proc Natl Acad Sci USA. 2012; 109(52): 21528–21533. doi: 10.1073/pnas.1215849110 23236133 10.1073/pnas.1215849110 PMC3535647

[9] Tran L, Greenwood-Van Meerveld B. Age-associated remodeling of the intestinal epithelial barrier. J Gerontol A BiolSci Med Sci. 2013; 68(9): 1045–1056. 10.1093/gerona/glt106 PMC3738030 23873964

[10] Clarke TB, Davis KM, Lysenko ES, Zhou AY, Yu Y, Weiser JN. Recognition of peptidoglycan from the microbiota by Nod1 enhances systemic innate immunity. Nat Med. 2010(2); 16: 228–231. doi: 10.1038/nm.2087 20081863 10.1038/nm.2087 PMC4497535

[11] Wu HJ, Wu E. The role of gut microbiota in immune homeostasis and autoimmunity. Gut Microbes. 2012; 3(1):4–14. doi: 10.4161/gmic.19320 22356853 10.4161/gmic.19320 PMC3337124

[12] Chen J, Wright K, Davis JM, Jeraldo P, Marietta EV, Murray J et al An expansion of rare lineage intestinal microbes characterizes rheumatoid arthritis. Genome Med. 2016; 8(1):43 doi: 10.1186/s13073-016-0299-7 27102666 10.1186/s13073-016-0299-7 PMC4840970

[13] Rogier R, Koenders MI, Abdollahi-Roodsaz S. Toll-like receptor mediated modulation of T cell response by commensal intestinal microbiota as a trigger for autoimmune arthritis. J Immunol Res. 2015; 2015: 527696. doi: 10.1155/2015/527696 10.1155/2015/527696 PMC4352938 25802876

[14] Zhang X, Zhang D, Jia H, Feng Q, Wang D, Liang D et al The oral and gut microbiomes are perturbed in rheumatoid arthritis and partly normalized after treatment. Nat Med. 2015; 21(8):895–905. doi: 10.1038/nm.3914 26214836 10.1038/nm.3914

[15] Wu X, He B, Liu J, Feng H, Ma Y, Li D et al Molecular Insight into Gut Microbiota and Rheumatoid Arthritis. Int J Mol Sci. 2016;17(3): 431 Review. doi: 10.3390/ijms17030431 27011180 10.3390/ijms17030431 PMC4813281

[16] Percival RS, Marsh PD, Challacombe SJ. Serum antibodies to commensal oral and gut bacteria vary with age. FEMS Immunol Med Microbiol. 1996; 15(1): 35–42. 8871114 10.1111/j.1574-695X.1996.tb00356.x

[17] Newkirk MM, Goldbach-Mansky R, Senior BW, Klippel J, Schumacher HR Jr, El-Gabalawy HS et al Elevated levels of IgM and IgA antibodies to Proteus mirabilis and IgM antibodiesto Escherichia coli are associated with early rheumatoid factor (RF)-positive rheumatoid arthritis. Rheumatology (Oxford). 2005; 44(11): 1433–1441. 16091399 10.1093/rheumatology/kei036

[18] Frulloni L, Lunardi C, Simone R, Dolcino M, Scattolini C, Falconi M. Identification of novel antibody associated with autoimmune pancreatitis. N Engl J Med. 2009; 361(22): 2135–2142. doi: 10.1056/NEJMoa0903068 19940298 10.1056/NEJMoa0903068

[19] Shiota S, Matsunari O, Watada M, Yamaoka Y. Serum Helicobacter pylori CagA antibody as a biomarker for gastric cancer in east-Asian countries. Future Microbiol. 2010; 5(12): 1885–1893. doi: 10.2217/fmb.10.135 21155667 10.2217/fmb.10.135 PMC3044821

[20] D'Souza AL. Ageing and the gut. Postgrad Med J. 2007; 83(975): 44–53. doi: 10.1136/pgmj.2006.049361 17267678 10.1136/pgmj.2006.049361 PMC2599964

[21] Singh H, Raghava GP. ProPred1: prediction of promiscuous MHC Class-I binding sites. Bioinformatics. 2003; 19(8):1009–1014. 12761064 10.1093/bioinformatics/btg108

[22] Saraswat A, Shraddha, Jain A, Pathak A, Verma SK, Kumar A. Immuno-informatic speculation and computational modeling of novel MHC-II human leukocyte antigenic alleles to elicit vaccine for ebola virus. J Vaccines Vaccin 3:141 doi: 10.4172/2157-7560.1000141

[23] Bhasin M., Garg A. and Raghava GPS. PSLpred: prediction of subcellular localization of bacterial proteins. Bioinformatics. 2005; 21(10): 2522–2524. doi: 10.1093/bioinformatics/bti309 15699023 10.1093/bioinformatics/bti309

[24] Greenbaum J, Sidney J, Chung J, Brander C, Peters B, Sette A. Functional classification of class II human leukocyte antigen (HLA) molecules reveals seven different supertypes and surprising degree of repertoire sharing across supertypes. Immunogenetics. 2011; 63(6): 325–335. doi: 10.1007/s00251-011-0513-0 21305276 10.1007/s00251-011-0513-0 PMC3626422

[25] Rigoni R, Grassi F, Villa A, Cassani B. RAGs and BUGS: An alliance for autoimmunity. Gut Microbes. 2016; 7(6): 503–511. doi: 10.1080/19490976.2016.1228517 27575988 10.1080/19490976.2016.1228517 PMC5153610

[26] Bonder MJ, Kurilshikov A, Tigchelaar EF, Mujagic Z, Imhann F, Vila AV et al The effect of host genetics on the gut microbiome. Nat Genet. 2016; 48(11):1407–1412. doi: 10.1038/ng.3663 27694959 10.1038/ng.3663

[27] López P, de Paz B, Rodríguez-Carrio J, Hevia A, Sánchez B, Margolles A et al Th17 responses and natural IgM antibodies are related to gut microbiotacomposition in systemic lupus erythematosus patients. Sci Rep. 2016; 6: 24072 doi: 10.1038/srep24072 27044888 10.1038/srep24072 PMC4820712

[28] Harmsen HJ, Pouwels SD, Funke A, Bos NA, Dijkstra G. Crohn's disease patients have more IgG-binding fecal bacteria than controls. Clin Vaccine Immunol. 2012; 19(4): 515–521. doi: 10.1128/CVI.05517-11 22336288 10.1128/CVI.05517-11 PMC3318288

[29] Shamriz O, Mizrahi H, Werbner M, Shoenfeld Y, Avni O, Koren O et al Microbiota at the crossroads of autoimmunity. Autoimmun Rev. 2016; 15(9): 859–869. doi: 10.1016/j.autrev.2016.07.012 27392501 10.1016/j.autrev.2016.07.012

[30] Shin NR, Whon TW, Bae JW. Proteobacteria: microbial signature of dysbiosis in gut microbiota. Trends Biotechnol. 2015; 33(9):496–503. Review. doi: 10.1016/j.tibtech.2015.06.011 26210164 10.1016/j.tibtech.2015.06.011

[31] Graessler J, Qin Y, Zhong H, Zhang J, Licinio J, Wong ML et al Metagenomic sequencing of the human gut microbiome before and after bariatric surgery in obese patients with type 2 diabetes: correlation with inflammatory and metabolic parameters. Pharmacogenomics J. 2013; 13(6): 514–522. doi: 10.1038/tpj.2012.43 23032991 10.1038/tpj.2012.43

[32] Mrozinska S, Radkowski P, Gosiewski T, Szopa M, Bulanda M, Agnieszka H et al Qualitative Parameters of the Colonic Flora in Patients with HNF1A-MODY Are Different from Those Observed in Type 2 Diabetes Mellitus. J Diabetes Res. 2016;2016: 3876764 10.1155/2016/3876764 PMC5078663 27807544

[33] Global Burden of Disease Study 2013 Collaborators. Global, regional, and national incidence, prevalence, and years lived with disability for 301 acute and chronic diseases and injuries in 188 countries, 1990–2013: a systematic analysis for the Global Burden of Disease Study 2013. Lancet. 2015; 386(9995):743–800. doi: 10.1016/S0140-6736(15)60692-4 26063472 10.1016/S0140-6736(15)60692-4 PMC4561509

[34] Wentworth JM, Naselli G, Brown WA, Doyle L, Phipson B, Smyth GK et al Pro-inflammatory CD11c+CD206+ adipose tissue macrophages are associated with insulin resistance in human obesity. Diabetes. 2010; 59(7): 1648–1656. doi: 10.2337/db09-0287 20357360 10.2337/db09-0287 PMC2889764

[35] Paschke A, Grzelka A, Zawada A, Zozulińska-Ziółkiewicz D Clinical characteristics and autoantibody pattern in newly diagnosed adult-onset autoimmune diabetes. Pol Arch Med Wewn. 2013; 123(7–8): 401–408. 23792409 10.20452/pamw.1831

[36] Packard TA, Li QZ, Cosgrove GP, Bowler RP, Cambier JC. COPD is associated with production of autoantibodies to a broad spectrum of self-antigens, correlative with disease phenotype. Immunol Res. 2013; 55(1–3): 48–57. doi: 10.1007/s12026-012-8347-x 22941590 10.1007/s12026-012-8347-x PMC3919062

[37] Somers K, Geusens P, Elewaut D, De Keyser F, Rummens JL, Coenen M et al Novel autoantibody markers for early and seronegative rheumatoid arthritis. J Autoimmun. 2011; 36(1): 33–46. doi: 10.1016/j.jaut.2010.10.003 21071175 10.1016/j.jaut.2010.10.003

[38] Morohoshi K, Patel N, Ohbayashi M, Chong V, Grossniklaus HE, Bird AC et al Serum autoantibody biomarkers for age-related macular degeneration and possible regulators of neovascularization. Exp Mol Pathol. 2012; 92(1): 64–73. doi: 10.1016/j.yexmp.2011.09.017 22001380 10.1016/j.yexmp.2011.09.017

