# Supplementary material for: Gut bacterial peptides with autoimmunity potential as environmental trigger for late onset complex diseases: In–silico study
Source: PLoS One. 2017 Jul 5;12(7):e0180518. doi: 10.1371/journal.pone.0180518 (PMC5498033; doi:10.1371/journal.pone.0180518)
Supplement: S1 Fig — (DOCX) [file pone.0180518.s001.docx]

**Supplementary figure 1:** HLA class II alleles showing significant difference in binding affinity between autoimmune candidate peptides and random peptide


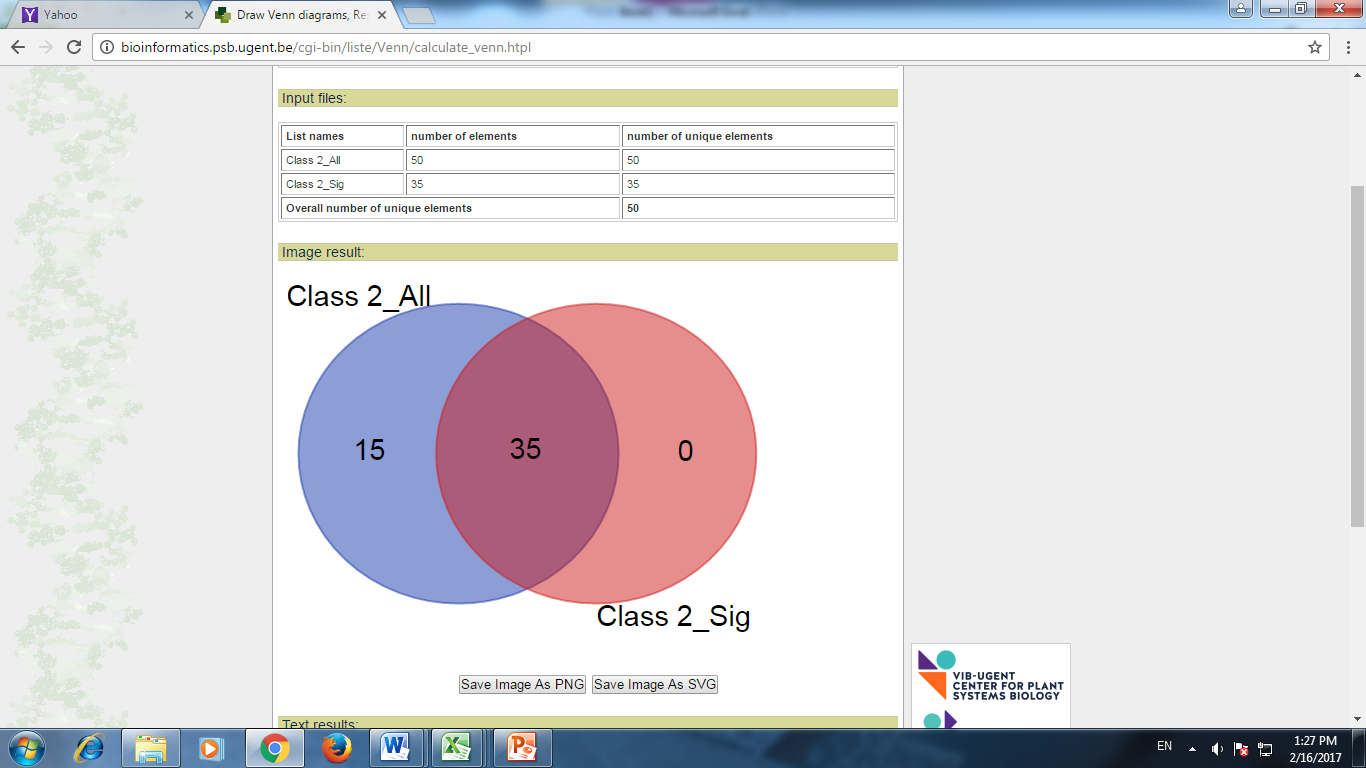


HLA_DRB1_1327 HLA_DRB1_0703 HLA_DRB1_1301 HLA_DRB1_1305 HLA_DRB1_0402 HLA_DRB1_0401 HLA_DRB1_1114 HLA_DRB1_1328 HLA_DRB1_0308 HLA_DRB1_0306 HLA_DRB1_0307 HLA_DRB1_0305

HLA_DRB1_1104 HLA_DRB1_0311 HLA_DRB1_1323 HLA_DRB1_0301 HLA_DRB1_1120 HLA_DRB1_1128

HLA_DRB1_0813 HLA_DRB1_1106 HLA_DRB1_0405 HLA_DRB1_0408 HLA_DRB1_0102 HLA_DRB1_0410

HLA_DRB1_1302 HLA_DRB1_1311 HLA_DRB1_1304 HLA_DRB1_1102 HLA_DRB1_0817 HLA_DRB1_0701 HLA_DRB1_0101 HLA_DRB1_1322 HLA_DRB1_1121 HLA_DRB1_0426 HLA_DRB1_1101

HLA_DRB5_0105 HLA_DRB1_0804 HLA_DRB1_0806 HLA_DRB1_1501 HLA_DRB5_0101 HLA_DRB1_0404 HLA_DRB1_1107 HLA_DRB1_0802 HLA_DRB1_0801

HLA_DRB1_1321 HLA_DRB1_1307 HLA_DRB1_0423 HLA_DRB1_1506 HLA_DRB1_1502 HLA_DRB1_0309
